# Implementing Diabetic Eye Care in Rural Primary Care Through Teleophthalmology and Pop-Up Clinics: Mixed Methods Study

**DOI:** 10.2196/106290

**Published:** 2026-09-03

**Authors:** Nisha G Arya, Victoria Koltchine, Talia C Gearinger, Sherianne Buehler, Reza Yousefi-Nooraie, Rajeev S Ramchandran

**Affiliations:** 1 University of Rochester School of Medicine and Dentistry, 601 Elmwood Avenue, Rochester, NY, 14642, United States, 1 5852752100; 2 Flaum Eye Institute, University of Rochester Medical Center, Rochester, NY, United States; 3 Environmental Medicine and Public Health Sciences, University of Rochester School of Medicine and Dentistry, Rochester, NY, United States; 4 Center for Community Health and Prevention, University of Rochester, Rochester, NY, United States

**Keywords:** teleophthalmology, mobile health units, diabetic retinopathy, implementation science, mixed methods research, Consolidated Framework for Implementation Research

## Abstract

**Background:**

Diabetic retinopathy is the leading cause of blindness in US adults. Fewer than half of patients with diabetes in rural areas complete the recommended screening that could prevent blindness. Store-and-forward teleophthalmology and mobile pop-up eye clinics have each been shown to improve screening access, but studies examining the implementation of both modalities within the same rural primary care system are lacking.

**Objective:**

This study aimed to identify multilevel facilitators and barriers to implementing teleophthalmology and pop-up eye clinics for diabetic eye care in 2 rural primary care clinics in upstate New York.

**Methods:**

We conducted a mixed methods implementation study with prospective semistructured interviews and retrospective, descriptive electronic health record data at 2 university-affiliated primary care clinics classified as “isolated rural,” guided by the Consolidated Framework for Implementation Research (CFIR). Electronic health record data from 885 adults with diabetes were used to characterize patient demographics and eye care use. Patients were categorized into 3 groups: eye exam with an eye doctor (n=586), on-site primary care–based eye exam (n=91; teleophthalmology n=39 and pop-up clinic n=52), or no recent eye exam (n=208). Semistructured interviews were conducted with 20 patients (n=5 per subgroup) and 14 staff members (primary care clinicians, optometrists, and administrative personnel), purposively sampled to capture diverse perspectives across eye care pathways and roles. Interviews were coded using CFIR domains and analyzed thematically until no new themes emerged.

**Results:**

Patients in the on-site primary care eye exam group were the most socially disadvantaged, with the lowest proportions reporting no transportation needs (42/91, 46.2%), no housing instability (28/91, 30.8%), and no food insecurity (36/91, 39.6%). Over 18 months, 58.4% (52/89) of scheduled pop-up clinic appointments were completed, and 92.3% (36/39) of teleophthalmology images were gradable. Pop-up clinics detected higher rates of diabetic retinopathy (11/52, 21.2% vs 3/39, 7.7%), cataract (29/52, 55.8% vs 3/39, 7.7%), and reduced visual acuity (23/52, 44.2% vs 11/39, 28.2%) than teleophthalmology. Qualitative analysis revealed that patients viewed both modalities as a convenient “one-stop shop” and valued trust in primary care staff. Staff identified complementary barriers: training and workflow complexity for teleophthalmology, and underutilization and scheduling challenges for pop-up clinics. Implementation champions across roles proposed an integrated workflow in which same-day teleophthalmology becomes a default component of annual diabetes visits, with targeted referral to pop-up clinics for patients with abnormal findings.

**Conclusions:**

Primary care–based eye programs in rural settings preferentially reached socially vulnerable patients with diabetes and detected substantial unmet eye pathology. However, operating teleophthalmology and pop-up clinics as separate programs led to inefficiencies in both programs. A stakeholder-driven integrated model embedding teleophthalmology into routine diabetes care, with targeted pop-up clinic referrals, may improve the reach, efficiency, and sustainability of diabetic eye screening in rural populations.

## Introduction

Diabetic retinopathy is the leading cause of blindness in working-age US adults, with higher prevalence in rural than urban populations [1,2]. Although annual dilated eye examinations can prevent blindness in more than 95% of patients with diabetes, fewer than half of patients in underserved rural areas complete this recommended screening [2-4]. Rural communities face compounding barriers to eye care access, including severe workforce shortages: nearly 63% of US counties have no practicing ophthalmologist, and rural ophthalmologist density is roughly one-tenth that of metropolitan areas [1]. These shortages are projected to worsen, with ophthalmology workforce adequacy in nonmetropolitan areas forecast to fall to 29% by 2035, compared with 77% in metropolitan areas [4]. Beyond workforce gaps, rural patients face geographic isolation, limited transportation, lower socioeconomic status, and higher rates of uninsurance, creating eye care deserts in which patients with diabetes are at elevated risk for undetected and untreated diabetic retinopathy [5-11].

Two delivery models have been developed to bring diabetic eye screening to patients in primary care settings. Store-and-forward teleophthalmology uses nonmydriatic retinal photography obtained by trained primary care staff, with the images transmitted to a remote eye care specialist for interpretation. Previous programs have demonstrated effectiveness in improving diabetic retinopathy detection and screening rates [5-10]. At the University of Rochester, teleophthalmology initially increased annual dilated eye exam rates from 34% to 75%, but camera use peaked in the first 4 months and then steadily declined; moreover, only 27% of patients completed recommended in-person follow-up after abnormalities were identified, with transportation barriers identified as a major contributor [11,12]. In contrast, mobile pop-up eye clinics bring equipment and clinical personnel to primary care offices, providing in-person dilated eye examinations that can address several limitations of teleophthalmology, including the inability to perform dilated fundus examinations and the gap between screening and follow-up. However, far less evidence exists on the effectiveness of mobile vision vans for diabetic retinopathy screening in the United States [13-15].

Although prior studies have examined either teleophthalmology or mobile clinics in isolation, no study has examined the implementation of both modalities coexisting within the same rural primary care system. Using the Consolidated Framework for Implementation Research (CFIR), this mixed methods study examined how patients, clinic staff, primary care clinicians, and managers experienced teleophthalmology and pop-up eye clinics at 2 primary care clinics classified as “isolated rural” (Rural-Urban Commuting Area [RUCA] code 10) in upstate New York to identify multilevel barriers and facilitators and to understand how eye care can be more effectively integrated into and sustained within rural primary care settings.

## Methods

### Study Design and Settings

This mixed methods convergent implementation study was conducted at 2 university-affiliated rural primary care clinics (clinics A and B) serving predominantly low-income populations in the greater Rochester, New York region. The convergent design involved 2 data strands collected in parallel and analyzed independently: prospective semistructured interviews served as the primary data source for identifying implementation barriers and facilitators, while retrospective descriptive electronic health record (EHR) data characterized the patient population and eye care use patterns [16]. The 2 strands were integrated during interpretation. This study adhered to the Good Reporting of a Mixed Methods Study (GRAMMS) guidelines (Checklist 1) and the Consolidated Criteria for Reporting Qualitative Research (COREQ) checklist (Checklist 2) [17,18].

The clinics are classified as RUCA code 10 under the United States Department of Agriculture RUCA classification, indicating that the clinics are in isolated rural regions where the primary commuting flow is outside of any census-defined urbanized area [19]. Both clinics provide comprehensive primary care to adults with chronic conditions, including large numbers of patients with diabetes, and historically had lower baseline rates of annual diabetic eye examinations compared to system-wide targets. In collaboration with the university-based ophthalmology department, 2 on-site eye care delivery models were implemented: store-and-forward teleophthalmology embedded within primary care visits and quarterly pop-up eye clinics providing in-person dilated examinations. These interventions were introduced as strategies to increase access, reduce travel burden, and reach socially vulnerable patients who were not completing recommended annual eye examinations.

### Clinic Workflow and Eye Care Programs

**Figure 1.**
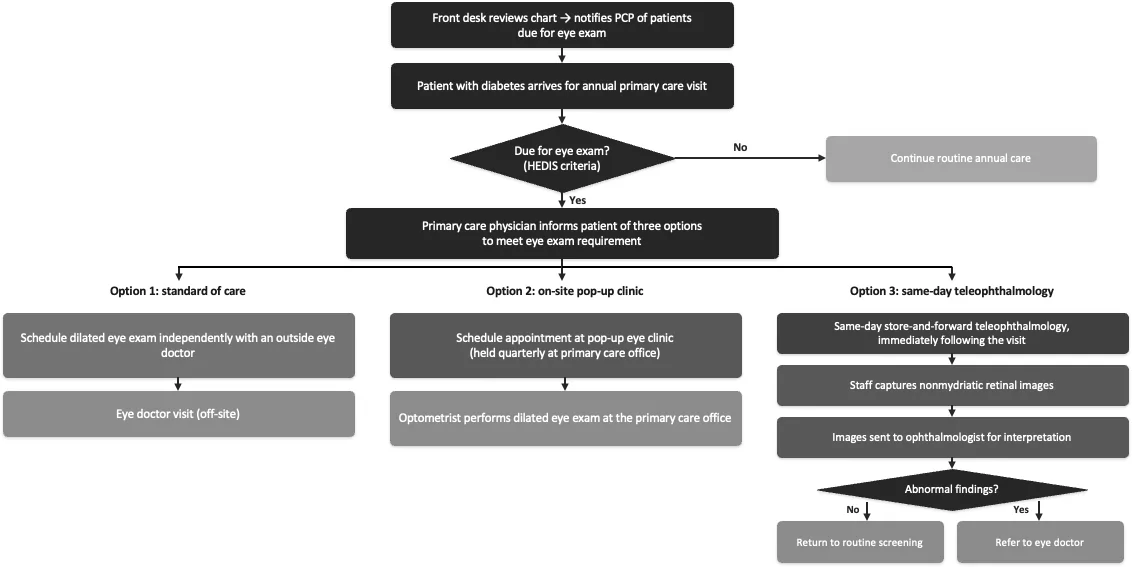
Current workflow. Options 2 and 3 are separate, unconnected pathways: no arrows link the pop-up eye clinic (option 2) and teleophthalmology (option 3) workflows, reflecting their current lack of integration. HEDIS: Healthcare Effectiveness Data and Information Set; PCP: primary care physician.

The current workflow for patients with diabetes due for an annual eye exam is represented in Figure 1. Front desk staff conduct previsit planning by reviewing incoming patients’ charts and notifying the primary care physician (PCP) of patients with diabetes who have not had an annual dilated eye exam in the previous year (Healthcare Effectiveness Data and Information Set [HEDIS] criteria for a diabetic eye exam [20]). At the primary care appointment, the PCP informs the patient of three options to meet their eye exam requirement: (1) independently schedule a dilated eye exam with an eye doctor, which represents the standard of care, (2) schedule an eye exam at the upcoming pop-up eye clinic held every 3 months at the primary care office, or (3) participate in store-and-forward teleophthalmology immediately following the appointment. Because options 2 and 3 are the on-site primary care–based interventions examined in this study, their workflows are described in detail below.

On the pop-up eye clinic day, a mobile vision van delivers eye exam equipment and personnel to an available room at the primary care office. A university-employed optometrist performs in-person comprehensive eye exams, including dilation to detect retinal and optic nerve diseases such as diabetic retinopathy, usually lasting around 45 minutes. Insurance billing for the pop-up eye clinic is done on the EHR platform in the same manner as at the university eye clinic. Ophthalmology and primary care staff both contact a list of patients with diabetes who need an eye exam to schedule them for an eye doctor appointment at the primary care clinic.

At a teleophthalmology visit, a trained primary care staff member assesses a patient’s vision using a standard Snellen chart and uses a nonmydriatic NW400 Topcon digital retinal camera to take photographs of the patient’s eyes. These images are sent to a university-based ophthalmologist for interpretation, who recommends the appropriate follow-up based on the findings from the teleophthalmology screening. These exams are billed to insurance using the 92,250 Current Procedural Terminology code [11].

### Quantitative Data Collection

We used EHR data from 885 adult patients diagnosed with diabetes mellitus who received care at clinics A and B to describe the population eligible for eye care services. Patients were included if they had at least one annual visit with their PCP at one of the 2 clinics between January 1, 2022, and June 30, 2024; were aged 18 years or older; and had a diagnosis of diabetes. Variables extracted included demographic characteristics (age, race, and ethnicity), insurance type, social needs (transportation, housing instability, and food insecurity), health behaviors (tobacco use), comorbidities (hypertension, blood urea nitrogen, hemoglobin A1c, and low-density lipoprotein [LDL] levels), and eye pathology findings from the on-site examination groups.

### Qualitative Data Collection

The CFIR guided both qualitative data collection and analysis [21]. For this study, four CFIR domains most relevant to the research questions were selected: (1) inner setting and individuals, (2) intervention characteristics, (3) implementation process, and (4) outer setting. Interview guides (Multimedia Appendix 1) and the coding framework were organized around these domains.

We conducted semistructured interviews with 20 patients with diabetes, sampling 5 from each of 4 subgroups: eye exam with an eye doctor, teleophthalmology, pop-up clinic, and no recent eye exam. Staff interviews were conducted with 8 clinic staff (2 PCPs and 2 staff members who had participated in teleophthalmology and pop-up eye clinics at each clinic) and 6 administrative staff (4 ophthalmology billing team members and 2 ophthalmology outreach program coordinators), for a total of 14 staff participants. No patient or staff member declined participation. All interviews were conducted by NGA, a medical student not involved in clinical care at the study sites, via telephone. Participants were informed that NGA was a medical student conducting research on eye care programs. Verbal consent was obtained, and all interviews were audio-recorded and professionally transcribed verbatim. Interviews lasted approximately 30 minutes. The interviewer recorded brief field notes after each interview to capture contextual observations and initial impressions; transcripts were not returned to participants for comment. Patients who were interviewed received US $25 for their participation. Interview guides were developed using these 4 CFIR domains to elicit perspectives on the implementation, integration, and sustainability of teleophthalmology and pop-up eye clinics (Multimedia Appendix 1).

### Data Analysis

Quantitative data from 885 adult patients diagnosed with diabetes mellitus who received care at clinics A and B were used to describe the population eligible for eye care services. These descriptive data were used to characterize the study population and patterns of eye care use; no statistical comparisons for hypothesis testing were performed. Patients were categorized into three groups based on documented eye care use: (1) those who received a dilated eye exam by an eye doctor, (2) those who received an on-site eye exam at their primary care clinic through teleophthalmology or a pop-up eye clinic, and (3) those who had no eye exam in at least the last 2 years. We summarized demographic characteristics (age, race, and ethnicity), insurance type, social needs (transportation, housing instability, and food insecurity), health behaviors (tobacco use), and comorbidities (hypertension, blood urea nitrogen, hemoglobin A1c, and LDL levels) using means, medians, and proportions (Table 1). For patients in the pop-up eye clinic and teleophthalmology groups, we also calculated attendance rates and the frequencies of eye pathologies identified (cataract, diabetic retinopathy, and reduced visual acuity).

**Table 1.** Demographic data by group.

Characteristic	Total, N=885	Exam with eye doctor group, n=586	On-site exam at primary care group^a^, n=91	No recent eye exam group^b^, n=208
Primary care clinic, n (%)				
Clinic A	466 (52.7)	290 (49.5)	63 (69.2)	113 (54.3)
Clinic B	419 (47.3)	296 (50.5)	28 (30.8)	95 (45.7)
Age (y), mean (SD)	65.1 (13.4)	68 (13.2)	62.5 (12.9)	58.1 (11.2)
Sex, n (%)				
Male	474 (53.6)	314 (53.6)	51 (56.0)	109 (52.4)
Female	411 (46.4)	272 (46.4)	40 (43.9)	99 (47.6)
Race, n (%)				
White	813 (91.9)	541 (92.3)	79 (86.8)	193 (92.8)
Black	46 (5.2)	27 (4.6)	11 (12.1)	8 (3.8)
Other^c^	20 (2.3)	13 (2.2)	1 (1.1)	6 (2.9)
Unknown	6 (0.7)	5 (0.9)	0	1 (0.5)
Hispanic ethnicity, mean (SD)	25 (2.8)	20 (3.4)	1 (1.1)	4 (1.9)
Insurance, n (%)				
Commercial	329 (37.2)	183 (31.2)	33 (36.3)	113 (54.3)
Medicare Advantage	399 (45.1)	307 (52.4)	40 (43.9)	52 (25)
Medicare	96 (10.8)	66 (11.3)	10 (10.9)	20 (9.6)
Commercially managed Medicaid	53 (5.9)	25 (4.3)	8 (8.8)	20 (9.6)
Medicaid	2 (0.2)	1 (0.2)	0 (0)	1 (0.5)
Unknown	6 (0.7)	4 (0.7)	0 (0)	2 (0.9)
Transportation needs, n (%)				
No needs	494 (55.8)	326 (55.6)	42 (46.2)	126 (60.6)
Unmet needs	24 (2.7)	17 (2.9)	3 (3.3)	4 (1.9)
Unknown	367 (41.5)	243 (41.5)	46 (50.6)	78 (37.5)
Housing instability, n (%)				
Low risk	406 (45.9)	277 (47.3)	28 (30.8)	101 (48.6)
High risk	36 (4.1)	22 (3.8)	6 (6.6)	8 (3.8)
Unknown	443 (50.1)	287 (48.9)	57 (62.6)	99 (47.6)
Food insecurity, n (%)				
No insecurity	467 (52.8)	317 (54.1)	36 (39.6)	114 (54.8)
Insecurity present	46 (5.2)	24 (4.1)	6 (6.6)	16 (7.7)
Unknown	372 (42.0)	245 (41.8)	49 (53.8)	78 (37.5)
Tobacco use status, n (%)				
Current smoker	125 (14.1)	66 (11.3)	21 (23.1)	38 (18.3)
Past smoker	359 (40.6)	243 (41.5)	40 (43.9)	76 (36.5)
Never smoker	394 (44.5)	273 (46.6)	29 (31.9)	92 (44.2)
Unknown	7 (0.8)	4 (0.7)	1 (1.1)	2 (0.9)
Hypertension^d^, n (%)	218 (24.6)	145 (24.7)	19 (20.9)	54 (25.9)
Median BUN^e^ (range) (mg/dL)	17 (13-22)	18 (14-22)	16 (11-21)	16 (13-20)
Median HbA1c (range)	7.1 (6.4-7.9)	7.1 (6.4-7.8)	7.1 (6.4-7.9)	7.1 (6.3-8.1)
LDL^f^ >100 mg/dL, n (%)	190 (21.5)	119 (20.3)	20 (21.9)	51 (24.5)

a Includes patients who received teleophthalmology or pop-up eye clinic exams.

b The patient did not meet Healthcare Effectiveness Data and Information Set (HEDIS) criteria for diabetic screening, defined as an eye exam every 2 years if there is no history of diabetic retinopathy, or an eye exam every year if there is a history of diabetic retinopathy.

c Includes American Indian, Alaska Native, and Asian.

d Systolic blood pressure >140 mm Hg or diastolic blood pressure >90 mm Hg, as defined by the American Diabetes Association.

e BUN: blood urea nitrogen.

f LDL: low-density lipoprotein.

Qualitative data were analyzed using reflexive thematic analysis as described by Braun and Clarke [22], informed by their 2023 recommendations for good practice in thematic analysis. The CFIR framework that guided interview development also served as a sensitizing framework during analysis, providing an initial organizing structure while allowing themes to be developed inductively from participants’ accounts.

Three authors (NGA, RY-N, and RSR) independently read all transcripts for familiarization. NGA generated initial codes line-by-line across the full dataset, with RY-N and RSR independently coding a subset of transcripts. The 3 coders met to compare codes, discuss discrepancies, and iteratively refine a shared codebook maintained in Excel. Candidate themes were developed by collating codes into broader patterns of meaning and then reviewed against the coded data and full transcripts to ensure coherence and distinctiveness. Themes were defined, named, and organized into the 4 CFIR domains. Coding continued until no new themes were identified across successive transcripts, suggesting thematic sufficiency.

Reflexivity was addressed as follows: the research team included members with backgrounds in ophthalmology (RSR), implementation science and qualitative methods (RY-N), and medical education (NGA). NGA, who conducted all interviews, was a medical student not involved in clinical care at the study sites, which may have facilitated candid responses from both patients and staff. RSR, as the ophthalmologist who helped develop the teleophthalmology and pop-up clinic programs, brought deep contextual knowledge but also a potential investment in the programs’ success; this was balanced by RY-N’s methodological expertise and independent perspective during coding and theme development. The team regularly discussed how their respective positions might shape their interpretation and actively sought disconfirming evidence during analysis.

Several strategies were used to enhance the trustworthiness of the qualitative findings. Credibility was supported through investigator triangulation (3 coders with different disciplinary perspectives), purposive sampling across 4 patient subgroups and multiple staff roles, and the use of direct participant quotations to ground interpretations in the data. Dependability was maintained through a documented audit trail including the evolving codebook, meeting notes from consensus discussions, and decision logs for theme refinement. Confirmability was addressed through team-based analysis, reflexive discussion of researcher positionality, and systematic comparison of themes across participant groups. Transferability was supported by thick description of the rural clinical setting, patient population demographics, and implementation context, enabling readers to assess the applicability of findings to similar settings.

### Current and Proposed Workflow Development

**Figure 2.**
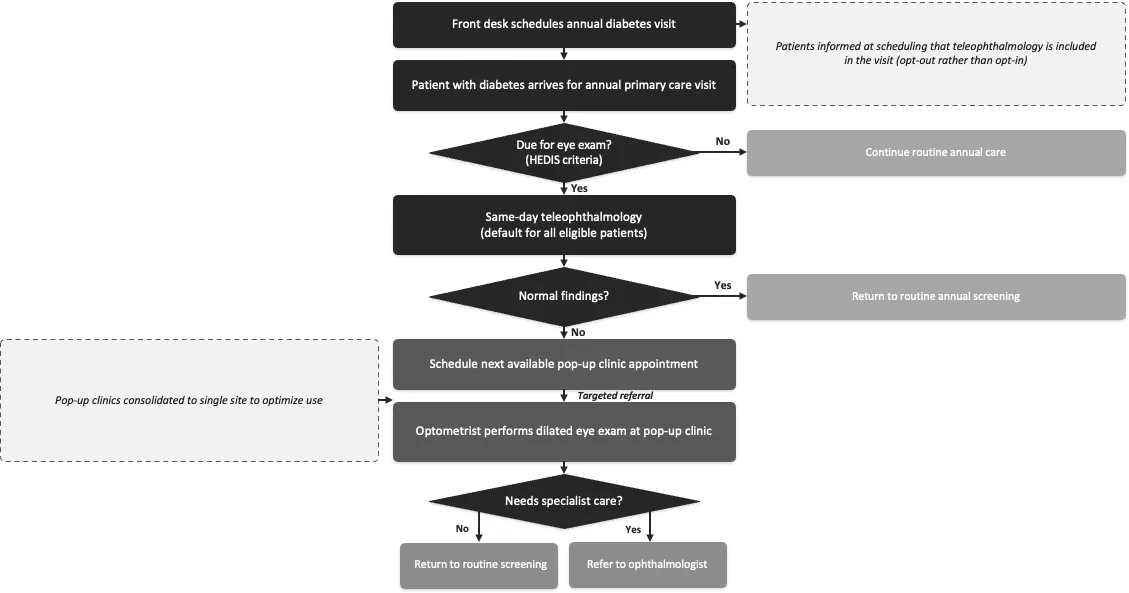
Proposed integrated workflow. HEDIS: Healthcare Effectiveness Data and Information Set.

Two workflow diagrams were developed as analytic outputs of this study. First, the current eye care workflow (Figure 1) was constructed by mapping the existing clinical processes described by staff and administrative participants during semistructured interviews, triangulated with operational documents and direct observation of clinic procedures [23]. Second, the proposed integrated workflow (Figure 2) was developed through an iterative, stakeholder-driven synthesis process [24]. During qualitative analysis, specific implementation solutions and process redesign suggestions were identified as a distinct category of codes within the CFIR implementation process domain. These stakeholder-generated recommendations (spanning clinical staff [primary care physicians, registered nurses, and licensed practical nurses], administrative personnel [front desk staff and practice managers], visiting optometrists, and financial administrators) were extracted, mapped to the barriers and facilitators they were intended to address, and synthesized into a unified process model. The proposed workflow was refined through discussion among the research team (RSR, RY-N, and NGA) to ensure internal consistency, alignment with the qualitative findings, and feasibility within the constraints identified by participants. The final model was reviewed by 2 participating stakeholders (1 practice manager and 1 optometrist) for face validity.

### Ethical Considerations

This study was approved by the Institutional Review Board of the University of Rochester (RSRB00009510). Verbal informed consent was obtained from all interview participants prior to participation. All interviews were deidentified, and audio recordings were stored securely in accordance with institutional data security policies. Patient participants received US $25 in compensation for their time. The research described adhered to the tenets of the Declaration of Helsinki [25].

## Results

### Eye Care Use

#### Patient Demographics

Of the 885 patients with diabetes, approximately equal proportions were seen at the 2 clinics (Table 1). Overall, 586 (66.2%) received a dilated eye examination by an eye doctor and met HEDIS criteria (n=290 at clinic A and n=296 at clinic B). Of the remaining 299 patients who did not meet HEDIS criteria, 91 (30.4%) received an on-site primary care–based eye exam through teleophthalmology (n=39) or pop-up clinic (n=52), while 208 (69.6%) had not received an eye exam in the last 2 years.

Of 885 patients, most were older (mean age 65.1, SD 13.4 y) and self-identified as White (n=813, 91.9%), reflecting the demographic composition of the rural catchment area. All had health insurance, most commonly Medicare Advantage (n=399, 45.1%). Nearly half of the patients reported social needs: only 55.8% (n=494) reported no transportation needs, 45.9% (n=406) were at low risk for housing instability, and 52.8% (n=467) reported no food insecurity (Table 1).

Table 1 shows the descriptive characteristics of the study population by eye care use group. Patients in the on-site primary care eye exam group (n=91) were the most socially disadvantaged, with the lowest proportions reporting no transportation needs (n=42, 46.2%), low risk for housing instability (n=28, 30.8%), and no food insecurity (n=36, 39.6%). This group also had the highest prevalence of current smoking (n=21, 23.1%) and past smoking (n=40, 43.9%). Patients in the no recent eye exam group (n=208) were younger (mean age 58.1, SD 11.2 y) and had the highest rate of commercial insurance (n=113, 54.3%) but also the highest rates of hypertension (n=54, 25.9%) and elevated LDL levels (n=51, 24.5%) compared with the other groups.

Within the on-site primary care–based eye exam group, patients in the pop-up clinic and teleophthalmology subgroups were similar with respect to age (mean 63.3, SD 14.0 y vs mean 61.5, SD 11.4 y), sex (29/52, 55.8% vs 22/39, 56.4% male patients), race (43/52, 82.7% vs 36/39, 92.3% White), no transportation needs (24/52, 46.2% vs 18/39, 46.2%), low risk of housing instability (19/52, 36.5% vs 9/39, 23.1%), no food insecurity (21/52, 40.4% vs 15/39, 38.5%), and current smoking status (12/52, 23.1% vs 9/39, 23.1%).

#### Program Use and Reach

Over the 18-month study period, 14 half-day pop-up eye clinics were held at 2 rural primary care clinics. Each session offered 8 appointment slots (112 total), of which 89 (79.5%) were scheduled and 52 (46.4% of total; 58.4% of scheduled) were completed. Among the 39 patients in the teleophthalmology group, adequate image quality was obtained for 36 (92.3%). In 3 (7.7%) patients, poor image quality prevented grading for diabetic retinopathy, and these patients were referred for an in-person eye exam with an eye doctor within 3 months. Taken together, the pop-up eye clinic and teleophthalmology programs provided eye care for 30.4% (91/299) of patients who were due for an annual diabetic eye exam but had not seen an eye doctor.

#### Eye Pathology

Among the 52 patients seen in the pop-up eye clinics, where a comprehensive dilated exam was performed by an optometrist, a higher rate of eye pathology was identified, including cataracts diagnosed in 29 (55.8%), diabetic retinopathy in 11 (21.2%), and reduced visual acuity worse than 20/40 in 23 (44.2%). No eye pathology was identified in 8 (15.4%) pop-up clinic patients.

Among the 39 patients who received a nonmydriatic screening exam via teleophthalmology, cataract and diabetic retinopathy were each identified in 3 (7.7%) patients, macular pathology in 2 (5.1%), glaucoma in 1 (2.6%), and reduced visual acuity in 11 (28.2%). No eye pathology was identified in 24 (61.5%) teleophthalmology patients.

### Facilitators of Implementation

Qualitative interviews with 20 patients and 14 staff members identified several facilitators of on-site eye care implementation (Table 2). Exact quotes can be found in Multimedia Appendix 2. Across all patient subgroups, proximity to the primary care office was the most frequently cited advantage, particularly for patients with transportation challenges: “It’s a long drive into the city, and I don’t drive anymore” [Teleophthalmology patient 5]. Closely related was the trust and familiarity that patients had developed with primary care staff over years of regular visits, which they contrasted with infrequent encounters at eye doctors’ offices: “They treat you like family, they don’t treat you like you’re just a patient” [Teleophthalmology patient 2].

**Table 2.** Summary of qualitative findings organized by Consolidated Framework for Implementation Research 2.0 domain.^a^

Theme	Barrier or facilitator	Brief finding
Individuals and inner setting		
Proximity of primary care office	Facilitator	Patients valued receiving eye care at a familiar, nearby location, reducing travel burden
Trust in primary care staff	Facilitator	Longstanding relationships with clinic staff increased willingness to participate
Assistance with scheduling referrals	Facilitator	Staff-assisted scheduling reduced patient burden of navigating follow-up appointments
Motivation and buy-in	Facilitator	Staff described the programs as meaningful and expressed pride in preventing vision loss
Limited staffing resources	Barrier	Short-staffing made it difficult to consistently assign trained personnel for teleophthalmology
Pop-up clinic underutilization	Barrier	Optometrists reported low patient volume at pop-up sessions relative to their regular clinics
Intervention characteristics		
“One-stop shop” integration	Facilitator	Patients preferred same-day, same-location eye care bundled with their diabetes visit
Patient perception of quality	Facilitator	Patients described on-site exams as thorough and comparable or superior to prior eye doctor visits
Continued need for glasses or specialty care	Barrier	Patients still required separate visits for eyeglasses and advanced eye disease management
Technical complexity of teleophthalmology	Barrier	Multistep camera and upload workflow created staff anxiety and inconsistent use
Implementation processes		
Workflow integration	Facilitator or barrier	Patients found same-day screening smooth and convenient, but lack of advance notification and competing transportation arrangements prevented some from participating
Advanced communication	Facilitator	Patients informed ahead of time were more willing to stay for same-day screening
Clinician-driven referral	Facilitator	Patients were more receptive when their primary care physician recommended on-site eye care
Implementation champions	Facilitator	Staff across roles proposed solutions and sustained program momentum despite challenges
Training gaps	Barrier	Only a subset of staff felt confident with the camera; errors caused delays
Coordination between staff	Barrier	Clinicians offered teleophthalmology inconsistently due to uncertainty about staff availability
Outer setting		
Insurance coverage	Mixed	Some patients had adequate coverage; others were uncertain about copays at on-site programs
Reimbursement disputes	Barrier	Billing disagreements with insurers over CPT^b^ codes created financial uncertainty

a Representative quotes for each theme are provided in Multimedia Appendix 2.

b CPT: Current Procedural Terminology.

Patients across modalities described on-site eye care as a “one-stop shop” that integrated diabetes care and eye screening into a single visit, reducing the burden of scheduling and traveling to a separate appointment. Several patients noted that staff assistance with scheduling follow-up referrals was particularly valuable, as navigating the referral process independently was difficult. Patients who had used either modality expressed high satisfaction with the quality of care, with some describing on-site exams as more thorough than previous eye doctor visits.

Among staff, motivation and buy-in were strong facilitators. Nurses described the programs as meaningful ways to prevent vision loss in patients who might otherwise “fall through the cracks,” and front desk staff felt motivated to coordinate follow-up despite the extra workload. Implementation champions spanning clinical and administrative roles, including registered nurses, licensed practical nurses, front desk staff, practice managers, optometrists, and a financial administrator, played a critical role in sustaining the programs and proposing solutions to operational challenges. Clinician endorsement was a key facilitator: patients were more receptive when the recommendation came from their primary care clinician.

### Barriers to Implementation

Several barriers limited the reach and efficiency of both programs (Table 2). For teleophthalmology, technical complexity was the most prominent barrier: staff described the camera workflow as involving many small steps, and missing a single step could prevent images from reaching the ophthalmologist. This was compounded by training gaps, with only a subset of staff at each clinic feeling confident operating the camera. One patient noted that their visit “took quite a while because neither of ’em knew how to do it” [Teleophthalmology patient 3]. Clinicians reported offering teleophthalmology inconsistently because of uncertainty about whether trained staff would be available, reflecting broader challenges with staff coordination.

For pop-up clinics, underutilization was the central barrier. Visiting optometrists reported that schedules were frequently unfilled, with 1 noting: “When I cancel to come to the pop-up clinic and there are only 2 patients on the schedule, it feels like a waste of time and money” [Optometrist 1]. Limited staffing resources affected both programs, as running eye care alongside routine primary care required pulling staff from other duties.

At the patient level, the continued need for eyeglasses and specialty care was identified as a limitation, as patients still had to travel elsewhere to purchase eyeglasses or receive treatment for complex eye disease. Regarding the outer setting, patients reported uncertainty about insurance coverage for on-site eye exams, and financial administrators described reimbursement disputes with insurers over Current Procedural Terminology code interpretation for teleophthalmology. Lack of advance communication about same-day teleophthalmology also led some patients to leave before screening because transportation arrangements had already been made.

### Stakeholder-Proposed Integrated Workflow

Based on stakeholder recommendations identified through qualitative analysis, an integrated workflow was developed that links teleophthalmology and pop-up clinics into a single care pathway (Figure 2). In this model, all primary care staff are trained to perform teleophthalmology, which is positioned as a default component of annual diabetes visits rather than an optional add-on. At scheduling, front desk staff inform patients with diabetes that same-day teleophthalmology will follow their primary care visit. This default approach normalizes staff allocation and addresses clinician uncertainty about staff availability, identified as a barrier in the qualitative findings. Patients with abnormal teleophthalmology findings are scheduled directly into the next pop-up clinic for a comprehensive dilated exam, creating a predictable referral stream that addresses pop-up clinic underutilization. Patients with normal results return to routine screening intervals. Consistent with practice managers’ recommendations, the model consolidates the 2 rural pop-up clinics into a single site to optimize optometrists’ time and clinic space.

## Discussion

### Interpretation of Findings and Implications for Implementation

This mixed methods convergent study examined multilevel barriers and facilitators to implementing teleophthalmology and pop-up eye clinics for diabetic eye care in 2 isolated rural primary care clinics. On-site eye care programs preferentially reached socially vulnerable patients, that is, those with the highest rates of transportation needs, housing instability, food insecurity, and tobacco use, and detected substantial unmet eye pathology, including diabetic retinopathy in 21.2% (11/52) of pop-up clinic patients. However, operating the 2 modalities as separate, parallel programs created complementary inefficiencies: staff lacked confidence and consistent workflow support for teleophthalmology, while pop-up clinic appointment slots were underutilized. Stakeholder champions proposed an integrated workflow linking the 2 modalities into a single care pathway, which is presented in the *Results* section.

The finding that on-site eye care disproportionately reached socially disadvantaged patients suggests these programs may function as equity-targeting strategies rather than solely as screening interventions. Patients across all groups described the primary care office as a trusted, familiar “one-stop shop,” reinforcing prior evidence that reducing travel burden and leveraging established primary care relationships facilitates screening engagement in rural communities [26,27]. The pop-up clinic diabetic retinopathy rate of 21.2% (11/52) is consistent with national estimates of 18% to 22% among US adults with diabetes [28,29]. The higher detection rates in pop-up clinics compared with teleophthalmology reflect the greater sensitivity of comprehensive dilated examinations for anterior segment disease and subtle retinal findings, rather than necessarily a difference in underlying disease burden [30,31].

The teleophthalmology diabetic retinopathy detection rate of 7.7% (3/39) was lower than the 11% reported in the prior University of Rochester teleophthalmology study using the same nonmydriatic approach, and lower than rates in other large-scale programs [11,32,33]. Beyond the small sample size, this may reflect inconsistent camera use by staff who lacked confidence with the technology. Leeman et al [11] reported that camera use peaked early and then declined steadily, and qualitative findings in the present study revealed similar patterns: staff described fear of “doing it wrong,” while champions argued that routine use would make it “second nature.” Infrequent use may reduce both screening volume and image quality, potentially lowering detection sensitivity.

A critical finding was the disconnect between patient and system perspectives: patients experienced teleophthalmology and pop-up clinics as a single way of “getting eye care at the primary care office,” while staff operationalized them as separate programs with distinct workflows. By examining these 2 coexisting modalities within the same rural primary care system, this study reveals how parallel implementation without integration creates complementary inefficiencies. The stakeholder-proposed integrated workflow addresses this tension by repositioning teleophthalmology as a default component of annual diabetes visits, shifting from an “opt-in” to an “opt-out” approach similar to the United Kingdom National Diabetic Eye Screening Programme [34]. This default design directly addresses 2 key barriers: clinician uncertainty about staff availability for teleophthalmology and failure to inform patients in advance about same-day screening. Linking abnormal findings to targeted pop-up clinic referrals creates a predictable referral stream that may improve pop-up use, which operated at only 58.4% (52/89) of scheduled capacity during the study period. Importantly, this model emerged from the convergent recommendations of frontline champions across clinical, administrative, and financial roles rather than from the research team, lending it practical credibility that may facilitate adoption. Economic evaluations support teleophthalmology’s value in rural populations when adequately integrated and scaled [35], and the proposed workflow may provide the integration structure needed to realize this potential.

This study has several limitations. It was conducted in 2 clinics within a single university-affiliated health system in 1 rural region, which may limit generalizability to settings with different patient populations, staffing models, or reimbursement structures. The predominantly White population (813/855, 91.9%) reflects the catchment area demographics but limits applicability to more racially and ethnically diverse communities. The quantitative component was intentionally descriptive and not powered for hypothesis testing, and the number of patients in the teleophthalmology (n=39) and pop-up (n=52) groups was modest. Twenty patients and 14 staff members were interviewed, which constrains the range of perspectives captured. No patient experienced both modalities, so comparisons were based on individual experience and hypothetical descriptions rather than direct within-person comparisons. The proposed integrated workflow requires prospective evaluation before its effectiveness can be established.

### Conclusions

Primary care–based eye programs in isolated rural settings can preferentially reach socially vulnerable patients with diabetes and detect substantial unmet eye pathology, positioning them as both screening and health equity interventions. However, implementing teleophthalmology and pop-up clinics as separate programs leads to predictable inefficiencies that threaten sustainability. The stakeholder-driven, integrated model that embeds teleophthalmology into routine diabetes care with targeted pop-up clinic referrals offers a pathway to align these complementary modalities. More broadly, this study illustrates that when multiple delivery innovations coexist within a single clinical setting, intentional integration, informed by the perspectives of frontline staff and patients, is essential to realize their combined potential. Future studies should evaluate whether this integrated workflow increases screening rates, staff proficiency, and follow-up completion and whether the model is transferable to other rural primary care systems.

## Supplementary material

10.2196/106290 Multimedia Appendix 1 Interview guide.

10.2196/106290 Multimedia Appendix 2 Representative quotes organized by Consolidated Framework for Implementation Research 2.0 domain and theme.

10.2196/106290 Checklist 1 GRAMMS checklist.

10.2196/106290 Checklist 2 COREQ checklist.

## Abbreviations

CFIR: Consolidated Framework for Implementation Research
COREQ: Consolidated Criteria for Reporting Qualitative Research
EHR: electronic health record
GRAMMS: Good Reporting of a Mixed Methods Study
HEDIS: Healthcare Effectiveness Data and Information Set
LDL: low-density lipoprotein
PCP: primary care physician
RUCA: Rural-Urban Commuting Area
