# Supplementary material for: Implementing Diabetic Eye Care in Rural Primary Care Through Teleophthalmology and Pop-Up Clinics: Mixed Methods Study
Source: JMIR Diabetes. 2026 Sep 3;11:e106290. doi: 10.2196/106290 (PMC13541231; doi:10.2196/106290)
Supplement: Supplementary file 1 [file diabetes-v11-e106290-s001.docx]

Interview Guide

**Patient Interviews**

Introduction

- Explain the purpose of the interview: to understand experiences and perspectives related to eye care programs (teleophthalmology, pop-up clinic, or standard referrals).
- Assure confidentiality and voluntary participation.
- Obtain verbal consent to record the interview.

Intervention Characteristics

1. In your own words, what is the [pop-up eye clinic/teleophthalmology program]?
2. How well did the program fit into your primary care clinic experience?
   - Did you experience any disruptions or changes in your usual routine?
3. How does this program compare to being referred to an eye doctor’s office?
   - What do you like and dislike about this program compared to others?

Outer Setting

4. What challenges, if any, did you face in accessing the program (e.g., transportation, scheduling, cost)?
5. How much did participating in this program cost for you? Did the cost influence your willingness to participate?
6. What information was provided to convince you that this program would address your needs?

Inner Setting

7. How did the staff at the clinic handle your participation in the program?
   - Did they address any problems you encountered?
   - How receptive did you feel the staff were to this program?
8. Did the staff seem confident and prepared to deliver this service?

Characteristics of Individuals

9. What motivated you to participate in this program?
10. How did this program affect your understanding of the importance of diabetic eye care?

Process

11. How clear was the information you received about the program?
12. Were follow-up steps explained to you, and did you feel they were easy to complete?
13. Would you participate in this program again? Why or why not?
14. What changes would make the program more helpful or convenient for you?

Closing Questions

15. Is there anything else you’d like to share about your experience with this program?
16. Do you have any additional suggestions for improving its implementation or delivery?

**Staff and Provider Interviews**

Introduction

- Explain the purpose of the interview: to understand experiences and perspectives related to eye care programs (teleophthalmology, pop-up clinic, or standard referrals).
- Assure confidentiality and voluntary participation.
- Obtain verbal consent to record the interview.

Intervention Characteristics

1. In your own words, what is the purpose of the [pop-up eye clinic/teleophthalmology program]?
2. How has the program been added to your clinic’s workflow?
   - Were many changes required to implement it?
3. How does this program compare to current standard referrals or other eye care initiatives?

Outer Setting

4. What external factors (e.g., patient needs, reimbursement, transportation) have influenced the success of this program?
5. How do patients seem to perceive and engage with the program?
6. How much does the program typically cost for patients, and do you think cost affects their participation?

Inner Setting

7. How has this program affected your clinic’s workflow and overall operations?
   - What resources (e.g., training, time, staff) have been most helpful for implementation?
   - What additional resources would improve its integration?
8. What level of buy-in have you observed among clinic staff?

Characteristics of Individuals

9. How confident do you feel in your ability to support this program?
10. How has this program influenced your understanding of and approach to diabetic eye care?

Process

11. What steps were taken to prepare for and implement the program?

- Were key stakeholders involved in planning?
- How could the implementation process have been improved?

12. What problems have arisen during the implementation, and how were they addressed?
13. What additional supports or materials would make implementation smoother?
14. How sustainable do you think this program is in the long term?

Closing Questions

15. Is there anything else you’d like to share about your experience with this program?
16. Do you have any additional suggestions for improving its implementation or delivery?
