# Supplementary material for: Implementing Diabetic Eye Care in Rural Primary Care Through Teleophthalmology and Pop-Up Clinics: Mixed Methods Study
Source: JMIR Diabetes. 2026 Sep 3;11:e106290. doi: 10.2196/106290 (PMC13541231; doi:10.2196/106290)
Supplement: Supplementary file 2 [file diabetes-v11-e106290-s002.docx]

Representative quotes organized by CFIR 2.0 domain and theme.

| **Theme** | **Supporting Quotes** |
| --- | --- |
| **Individuals and Inner Setting** | |
| Proximity of primary care office | - “It's a long drive into the city, and I don't drive anymore. I have to wait for my daughter to take me, and she works.” [Teleophthalmology patient 5] - “I know I should go, but between work and taking care of my daughter who needs a lot of care, it just keeps getting pushed off.” [No eye exam patient 1] - “Just the convenience and, you know, you're already familiar with the office, so it was nice to be able to just go there and schedule some appointments.” [Pop-up clinic patient 1] - “I’d rather go to my primary care office because I don't want to drive all the way to the city which takes half an hour, but [Clinic B] is only about five minutes.” [Exam at eye doctor patient 5] |
| Trust in primary care staff | - “Oh, yeah, the staff were very professional.” [Pop-up clinic patient 1] - “I like 'em there. They treat you like family, they don't treat you like you're just a patient.” [Teleophthalmology patient 2] - “A pro I would say is we're familiar with most people there.” [Exam at eye doctor patient 2] - “The current staff in [Clinic B], I like them…I'm talking to the receptionists, the people in the front, the nurses are great.” [Exam at eye doctor patient 5] |
| Assistance with scheduling referrals | - “The receptionist helped me make my follow up appointment for me…instead of having to call myself.” [Pop-up clinic patient 3] - “The front desk set up for me to see a specialist. That was more convenient. I'm scatterbrained half the time, so I forget stuff.” [Teleophthalmology patient 5] |
| Motivation and buy-in | - “We did eye exams for people who have never had an eye exam before. It feels good to know we're catching things that would have been missed.” [LPN, Clinic A] - “The camera was difficult at first. But not so bad when you get used to it…And it helps all those people who would never get an eye exam otherwise.” [RN, Clinic B] - “Learning to use the camera was cool…even if it gets glitchy at times.” [Front desk staff, Clinic A] - “The whole staff needs to get on board with this. We either do it or we don’t. Why not do it if it helps all those people save their eyes?” [Practice manager, Clinic A] - “Setting up follow up appointments is the hardest part. We do it because patients really like it, but it does take time to make sure there is availability between the ophthalmology department and the patient.” [Front desk staff, Clinic B] |
| Limited staffing resources | - “It is not always possible to find a staff member who is trained in doing the pictures.” [Primary care physician, Clinic B] - “When we run short-staffed, we have to pull a person to operate the camera.” [Practice manager, Clinic A] |
| Pop-up clinic underutilization | - “I usually see 15-18 patients at my regular clinic. When I cancel to come to the pop-up clinic and there are only two patients on the schedule, it feels like a waste of time and money.” [Optometrist 1 at pop-up eye clinic] - “There are a lot of unfilled appointments in the pop-up clinic schedule. The room is just sitting there empty, and I am waiting around.” [Optometrist 2 at pop-up eye clinic] |
| **Intervention Characteristics** | |
| "One-stop shop" integration | - “I just walked down the hall after my appointment. It couldn't have been easier. And I ordered my glasses right after and called it a day...It was perfect.” [Pop-up clinic patient 2] - “I'm glad I didn't have to go anywhere else to be honest... it's just sometimes hard to get there because I don't drive right now. And so when [my primary care doctor] said I could have it done right there today, I'm like, oh yeah, I'll do it right now then.” [Teleophthalmology patient 2] - “If it was scheduled the same time as my doctor's appointment, then I'd be alright with it. I mean, I gotta go there anyway” [No eye exam patient 3] - “My mother is elderly and needs help, so she would rather be able to go get everything done at one office…a one-stop shop, I guess you can call it.” [Exam at eye doctor patient 4] - “A one stop shop would be great... It would be nice to be able to go to see the eye doctor right then and there when I need to have someone look at my diabetes.” [Exam at eye doctor patient 1] |
| Patient perception of quality | - “I thought they were more thorough with their exam than what I have had at [my optometrist].” [Pop-up clinic patient 1] - “She did a nice job. I mean she really checked and double checked and went and looked even further... I have no complaints whatsoever.” [Pop-up clinic patient 2] - “Oh, yeah. She knew what she was doing.” [Teleophthalmology patient 2] |
| Continued need for glasses/specialty care | - “The only difference is unless she has a whole truckload of eyeglasses... I got to go back to a place that sell glasses anyway, so it's almost like a double trip for me.” [Exam at eye doctor patient 2] - “Unless the diabetes exam is set up for, you know, you get your glasses, the whole nine yards there, then there would be no sense in me going to one twice, you know what I'm saying?” [Pop-up clinic patient 5] - “I can’t get my eyes checked at the primary care office because I need to see a special eye doctor because my eyes have a lot of problems.” [Exam at eye doctor patient 4] |
| Technical complexity of teleophthalmology | - “Operating the camera looked hard, but it is not so bad when you get down to it.” [LPN, Clinic A] - “You got to work the camera and understand how to upload the pictures. There are so many clicks and screens…if you miss one, you have to start over. If I do it often enough, I will remember all the steps.” [RN, Clinic B] |
| **Implementation Processes** | |
| Workflow integration | - “It was easy, it was quick… there were no issues. Everything went smoothly.” [Pop-up clinic patient 5] - “I would definitely do it again. That was the easiest fricking thing ever.” [Teleophthalmology patient 2] - “I mean, that [TO] being where I could go and it's right there. It's right close.” [No eye exam patient 2] - “If patients don’t know that they are having a picture taken, they leave. They say they’re too busy to wait an extra 10 minutes.” [Front desk staff 2] |
| Advanced communication | - “I would have stayed for the eye pictures…but I didn’t want to miss my ride from my daughter.” [No eye exam patient 4] - "My ride was already waiting in the parking lot. If I had known ahead of time, I could have planned for it." [No eye exam patient 3] |
| Clinician-driven referral | - “When I call to schedule patients for the camera or [the pop-up clinic], they only are interested when their primary care doctor tells them they need to do it, not me.” [Front desk staff 2] - “I don’t offer teleophthalmology to all diabetic patients…sometimes the nurses are too busy to take pictures, and it’s too hard for me to figure out if they are available or not.” [Primary care physician 2] |
| Implementation champions | - “There are so many patients that fall through the cracks and somehow don’t see an eye doctor for years, even if they come to their annual PCP appointments… If we just did it for every diabetic patient, it would become second nature.” [RN, Clinic B] - “I offered to train to use the camera during COVID when we did not have enough staff to keep the program running.” [LPN, Clinic A] - “You cannot estimate the utility of a program by financials alone. This isn't about the bottom line: it's about making sure these patients don't go blind. These outreach clinics serve an important mission of our medical center to serve the community.” [Financial administrator 1] - “[Clinic A] and [Clinic B] are only ten minutes apart…why do both do [pop-up eye clinics]? Is it really needed?” [Practice manager at Clinic A] - “It doesn't make sense to have two half-empty clinics ten minutes down the road from each other. Why not put them together and send the ones who need a closer look from the camera over to the eye doctor at the pop-up?” [Practice manager at Clinic B] |
| Training gaps | - “It takes a while to learn the websites that you have to put the photos in…if you miss one thing, it messes everything else up and doesn’t transfer to the doctor.” [LPN, Clinic A] - “There are so many little steps…I think my own barriers are myself and my confidence in making sure I’m doing it right and not wanting to snag on everything.” [RN, Clinic B] - “Taking the pictures and uploading them to the website only takes a couple minutes if you know what you are doing.” [Practice manager, Clinic A] - “The pictures took quite a while because neither of 'em knew how to do it.” [Teleophthalmology patient 3] |
| Coordination between staff | - “I think every member of the staff needs to be on board that eye screening should be offered here. There is too much confusion right now.” [Practice manager 2] |
| **Outer Setting** | |
| Insurance coverage | - “Part of the eye exam covered by Medicare and then the optometrists tell me how much I got to pay…but at [Clinic B], there's some confusion...with the doctors.” [Teleophthalmology patient 3] - “No, cost wouldn’t be an issue. I have coverage.” [Exam at eye doctor patient 4] |
| Reimbursement disputes | - “There were lots of billing issues at first. We have been working on reimbursements due to disagreements with insurance companies of what falls under the CPT 92250 definition.” [Financial administrator 1] - “The ability to participate is based on staffing, building the client base, and making sure our CPT billing was compliant…It was a slow boat to get everyone on board.” [Financial administrator 2] |
