# Supplementary material for: Implementing Diabetic Eye Care in Rural Primary Care Through Teleophthalmology and Pop-Up Clinics: Mixed Methods Study
Source: JMIR Diabetes. 2026 Sep 3;11:e106290. doi: 10.2196/106290 (PMC13541231; doi:10.2196/106290)
Supplement: Supplementary file 3 [file diabetes-v11-e106290-s003.docx]

**GRAMMS Checklist (Good Reporting of A Mixed Methods Study)**

1. **Describe the justification for using a mixed methods approach to the research question**
   a. Location in manuscript: Study Design and Settings (in Methods)
   b. Response: Qualitative interviews were needed to identify multilevel barriers and facilitators to implementation, while quantitative EHR data were needed to characterize the patient population and eye care utilization patterns. Neither data source alone could address the research questions.
2. **Describe the design in terms of the purpose, priority, and sequence of methods**
   a. Location in manuscript: Study Design and Settings (in Methods)
   b. Response: Convergent mixed methods design. Qualitative interviews (primary data source) and retrospective descriptive EHR data (secondary) were collected in parallel, analyzed independently, and integrated during interpretation.
3. **Describe each method in terms of sampling, data collection, and analysis**
   a. Location in manuscript: Quantitative Data Collection, Qualitative Data Collection, Data Analysis (in Methods)
   b. Response:
      i. Quantitative: EHR data from 885 adults with diabetes; descriptive statistics (means, medians, proportions); no hypothesis testing.
      ii. Qualitative: purposive sampling of 20 patients (5 per subgroup) and 14 staff, semistructured telephone interviews, and reflexive thematic analysis informed by CFIR.
4. **Describe where integration has occurred, how it has occurred, and who has participated in it**
   a. Location in manuscript: Study Design and Settings (in Methods), Discussion
   b. Response: Integration occurred during interpretation. Quantitative findings (who was reached, pathology detected, utilization rates) were linked with qualitative findings (how patients and staff experienced implementation) to develop a comprehensive understanding of implementation and to inform the stakeholder-proposed integrated workflow. All three analysts (NA, RYN, RR) participated in integration.
5. **Describe any limitation of one method associated with the presence of the other method**
   a. Location in manuscript: Discussion (Limitations paragraph)
   b. Response: The quantitative component was intentionally descriptive and not powered for hypothesis testing. The modest sample sizes in the teleophthalmology (n=39) and pop-up (n=52) groups limit the precision of quantitative estimates. Qualitative findings are based on 34 interviews, which constrains the range of perspectives captured.
6. **Describe any insights gained from mixing or integrating methods**
   a. Location in manuscript: Discussion
   b. Response: Integration revealed a critical disconnect that while quantitative data showed that on-site programs preferentially reached socially vulnerable patients, qualitative data revealed that staff operationalized the two modalities as separate programs despite patients experiencing them as unified. This insight directly informed the stakeholder-proposed integrated workflow.
