# Supplementary material for: Implementing Diabetic Eye Care in Rural Primary Care Through Teleophthalmology and Pop-Up Clinics: Mixed Methods Study
Source: JMIR Diabetes. 2026 Sep 3;11:e106290. doi: 10.2196/106290 (PMC13541231; doi:10.2196/106290)
Supplement: Supplementary file 4 [file diabetes-v11-e106290-s004.docx]

**COREQ Checklist (Consolidated Criteria for Reporting Qualitative Research)**

**Domain 1: Research Team and Reflexivity**
**Personal Characteristics**

1. Interviewer/facilitator: NA conducted all interviews.
   a. [Location: Qualitative Data Collection]
2. Credentials: BA (medical student at time of study)
   a. [Location: Title page]
3. Occupation: Medical student
   a. [Location: Title page / Author Contributions]
4. Gender: Female
   a. [Location: Not explicitly stated]
5. Experience and training: NA has a BA in anthropology during which she learned qualitative methods. In addition, she was mentored by RYN (PhD, implementation science and qualitative methods expertise). Interview guides were co-developed with RYN and RR.
   a. [Location: Qualitative Data Collection, Data Analysis]

**Relationship with Participants**

6. Relationship established: No prior relationship with participants. NA was not involved in clinical care at the study sites.
   a. [Location: Data Analysis in Reflexivity paragraph]
7. Participant knowledge of the interviewer: Participants were informed that NA was a medical student conducting research on eye care programs.
   a. [Location: Add to Qualitative Data Collection if not present]
8. Interviewer characteristics: NA was a medical student not involved in clinical care at the study sites, which may have facilitated candid responses.
   a. [Location: Data Analysis in Reflexivity paragraph]

**Domain 2: Study Design**
**Theoretical Framework**

9. Methodological orientation and theory: Reflexive thematic analysis (Braun and Clarke) informed by the Consolidated Framework for Implementation Research (CFIR).
   a. [Location: Data Analysis]

**Participant Selection**

10. Sampling: Purposive sampling across four patient subgroups (5 per group) and multiple staff roles.
    a. [Location: Qualitative Data Collection]
11. Method of approach: Patients and staff were contacted by telephone.
    a. [Location: Qualitative Data Collection]
12. Sample size: 20 patients and 14 staff members (34 total).
    a. [Location: Qualitative Data Collection]
13. Non-participation: No patient or staff declined participation.
    a. [Location: Qualitative Data Collection]

**Setting**

14. Setting of data collection: Telephone interviews.
    a. [Location: Qualitative Data Collection]
15. Presence of non-participants: Not applicable (telephone interviews).
    a. [Location: N/A]
16. Description of sample: 20 patients (5 from each of 4 eye care pathway subgroups) and 14 staff (8 clinic staff, 6 administrative staff).
    a. [Location: Qualitative Data Collection]

**Data Collection**

17. Interview guide: Developed using CFIR domains; provided in Appendix 1.
    a. [Location: Qualitative Data Collection]
18. Repeat interviews: No repeat interviews were conducted.
    a. [Location: N/A]
19. Audio/visual recording: All interviews were audio-recorded.
    a. [Location: Qualitative Data Collection]
20. Field notes: yes.
    a. [Location: Qualitative Data Collection]
21. Duration: 30 minutes
    a. [Location: Qualitative Data Collection]
22. Data saturation: Coding continued until no new themes were identified across successive transcripts, suggesting thematic sufficiency.
    a. [Location: Data Analysis]
23. Transcripts returned: transcripts were not returned to participants for comment.
    a. [Location: Qualitative Data Collection]

**Domain 3: Analysis and Findings**

**Data Analysis**

24. Number of data coders: Three (NA, RYN, RR).
    a. [Location: Data Analysis]
25. Description of the coding tree: Initial coding framework based on CFIR domains, iteratively refined. Codebook maintained in Excel.
    a. [Location: Data Analysis]
26. Derivation of themes: Themes developed through six-phase reflexive thematic analysis; both deductive (CFIR-informed) and inductive.
    a. [Location: Data Analysis]
27. Software: Microsoft Word documents with shared codebook in Excel. No dedicated qualitative software used.
    a. [Location: Data Analysis]
28. Participant checking: The proposed workflow was reviewed by two participating stakeholders for face validity.
    a. [Location: Current and Proposed Workflow Development]

**Reporting**

29. Quotations presented: Yes, representative quotations presented in text and in Supplementary Table 1.
    a. [Location: Results in Facilitators and Barriers sections; Supplementary Table 1]
30. Data and findings consistent: Yes, themes are supported by quotations and triangulated with quantitative findings.
    a. [Location: Results, Discussion]
31. Clarity of major themes: Four CFIR-informed domains with specific themes identified under each; summary in Table 2.
    a. [Location: Results, Table 2]
32. Clarity of minor themes: Minor themes (e.g., cost uncertainty, continued need for eyeglasses) presented within barrier/facilitator sections.
    a. [Location: Results in Barriers section]
